# Metagenome-assembled genomes from the gut microbiome of spontaneous diabetic macaques provide insights into microbes associated with type 2 diabetes mellitus

**DOI:** 10.1186/s12866-026-04902-2

**Published:** 2026-03-04

**Authors:** Yuchen Xie, Rui Wang, Xu Liu, Qiao Du, Shan Mo, Qinghua Liu, Guang Yang, Zhenxin Fan, Jing Li

**Affiliations:** 1 Key Laboratory of Bio-Resources and Eco-Environment (Ministry of Education), College of Life Sciences, Sichuan University, Chengdu, China; 2 Animal Experimental Center, West China Hospital, Sichuan University, Chengdu, China; 3 Ministry of Education Key Laboratory of Birth Defects and Related Diseases of Women and Children, Department of Pediatrics, West China Second University Hospital, Sichuan University, Chengdu, China; 4 Sichuan Key Laboratory of Development and Application of Monkey Models for Human Major Disease, Sichuan, China; 5 SCU-SGHB Joint Laboratory on Non-Human Primates Research, Meishan, China

**Keywords:** Type 2 diabetes mellitus, Metagenome-assembled genomes, *Macaca mulatta*, Lachnospiraceae, *Eubacterium*

## Abstract

**Background:**

Gut microbiota plays a crucial role in type 2 diabetes mellitus (T2DM) pathogenesis. Spontaneous T2DM macaques offer a valuable model for investigating contributions of gut microbiota to T2DM pathogenesis due to physiological similarities to humans and the absence of glucose-lowering drug interference.

**Results:**

We performed de novo assembly of metagenome-assembled genomes (MAGs) to explore the diversity and function of the gut microbiome at the genome level. We obtained 317 non-redundant MAGs from fecal metagenomes of macaques and 325 MAGs from humans, 168 of which were potential novel species. Most members of Lachnospiraceae, the main carriers of carbohydrate-active enzymes (CAZymes) and virulence genes, significantly increased in the guts of T2DM macaques and unmedicated T2DM patients. Further analysis on the MAGs of Lachnospiraceae identified concordant enrichment of potential microbial signatures of T2DM, including the macaque-derived *Eubacterium_Q sp900314445* (Mm_bin23) and human-derived *Eubacterium_F sp003491505* (Hs_bin20) and *Eubacterium ramulus* (Hs_bin147). They all carried intestinal barrier-associated virulence genes and diabetes-associated hypervirulence genes, which might be associated with barrier dysfunction, inflammation, and disrupt glucose homeostasis, thereby potentially contributing to the pathogenesis of T2DM.

**Conclusions:**

This study assembled extensive MAGs from the gut microbiome of spontaneous T2DM macaques and asymptomatic controls. Furthermore, we identified three *Eubacterium* genomes harboring virulence genes and diabetes-associated genes, which were significantly enriched in both T2DM macaques and T2DM humans, highlighting the potential roles of these microbes in T2DM pathogenesis. Overall, this study provides a critical foundation for elucidating gut microbiome-mediated mechanisms and developing targeted therapeutic strategies for T2DM.

**Supplementary Information:**

The online version contains supplementary material available at 10.1186/s12866-026-04902-2.

## Background

Type 2 diabetes mellitus (T2DM) is one of the most complex diseases and poses a formidable challenge to global public health [1, 2]. Accumulating evidence highlights the crucial role of gut microbiota in the development of T2DM [3–5]. Multiple mechanisms are implicated in gut microbiota-mediated pathogenesis, including barrier impairment, insulin resistance mediation, and mitochondrial dysfunction [6–8]. Although gut microbiota is highly susceptible to pharmaceutical interventions [9, 10], it is still difficult to conduct continuous studies without drug interference in patients. Human studies have struggled to standardize confounding factors such as diet, exercise, and antibiotic use. Most human T2DM patients receive long-term glucose-lowering medications (e.g., metformin), which directly alter gut microbiota composition and function, masking disease-associated microbial signatures [11, 12]. Difficulty in controlling for pharmaceutical exposures and other confounders in human studies potentially obscures microbial etiological factors, protective mechanisms, and diagnostic markers [13]. Investigating specific compositional and functional shifts in the gut microbiota during the development of T2DM requires minimizing the confounding effects of medications. Consequently, anti-diabetic medication-free animal models are critical in T2DM research.

With anatomical, developmental, and physiological similarities to humans, the rhesus macaque (*Macaca mulatta*) generally serves as an invaluable experimental animal for elucidating pathogenic mechanisms, validating therapeutic interventions, and modeling complex disorders [14, 15]. In particular, the spontaneous T2DM macaques, untreated with glucose-lowering drugs or antibiotics under strictly controlled conditions, can reveal microbiota dysbiosis driven purely by disease progression. Previous studies indicated that spontaneous T2DM macaques had inflammation, insulin resistance, gut microbiota imbalance, and fatty acid metabolism disorders, which were highly similar to the pathological mechanism of human T2DM [16–18]. However, the availability of spontaneous T2DM macaques is very limited. Wang et al. [19] identified only nine diabetic macaques among 2,000 screened. Our prior study found merely seven diabetic cases in 1,408 macaques [20] and eight cases in 1,698 macaques [16]. At present, there are still very limited studies on the gut microbiota of spontaneous T2DM macaques.

Furthermore, conventional metagenomic short-read taxonomic annotation frequently fails to capture significant microbial taxa due to database and algorithm limitations [21]. Genome-level functional exploration via metagenomic binning addresses these limitations by reconstructing genomes from low-abundance community members. Metagenome-assembled genomes (MAGs) from gastrointestinal tracts have substantially advanced microbiome diversity and function understanding. For instance, metabolic modeling based on MAGs derived from different T2DM disease groups identified metabolites associated with insulin resistance and elevated T2DM risk, demonstrating that the metabolic capacity of gut communities critically modulates disease progression [22]. MAGs derived from chronic diarrheal macaques and asymptomatic individuals strengthened the functional understanding of the macaque gut microbiome at the genome level, in particular chronic diarrhea individuals [23]. Tang et al. [24] identified 54 differentially distributed high-quality MAGs that could effectively distinguish diabetic kidney disease patients for early detection and stratification of disease severity to guide interventions. MAG assemblies could yield novel draft genomes, elucidating characteristics and functions of previously unculturable microorganisms.

Most studies on the T2DM human microbiome are potentially confounded by medication use, while prevailing analyses relying on read-based profiling often lack the resolution for genome-level comparisons. Spontaneous T2DM macaques that have not received any glucose-lowering drugs provide a valuable experimental model. Based on the previously identified spontaneous T2DM macaques, over 600 MAGs were assembled and reconstructed from macaque and human cohorts. This study aims to characterize the diversity and function of gut microbiome at the genome level and identify T2DM-associated microbes in both macaques and humans. We further identified concordant enrichment of specific Lachnospiraceae members, three *Eubacterium* MAGs carrying intestinal barrier-associated virulence genes and hypervirulence genes in both species. Our findings provide a clearer understanding of macaque gut microbiome dynamics, particularly in spontaneous T2DM, and offer novel insights into T2DM pathogenesis and host-microbe interactions.

## Methods

### Sample collection

The 10 fecal metagenomes of macaques (5 spontaneous T2DM macaques and 5 asymptomatic macaques) (Table S1) were retrieved from our previous study (GSA: CRA013607) [16]. 20 human gut metagenomes data, in which 10 T2DM patients were downloaded from PRJNA361402 [25], and 10 asymptomatic humans were downloaded from PRJNA638403 [26]. The experimental macaques used in this study were provided by Greenhouse Biotechnology Co., LTD (Meishan, China). All macaques were individually housed with standardized diets and environmental controls, following animal welfare guidelines throughout the sample collection process. This study was approved by the Ethics Committee of the College of Life Sciences, Sichuan University, and conducted by the local legislation and institutional requirements (Permit Number: SCU210429001).

### Co-assembly and binning of metagenome-assembled genomes

To co-assemble T2DM and asymptomatic samples, we employed MEGAHIT [27] with parameters -continue -kmin-1pass -k-list 27,37,47,57,67,77,87 -min-contig-len 1000. Genome reconstruction was conducted through MetaBAT2 [28] binning (-minContigDepth 2 -minContigLength 2000). Raw bins derived from de novo assemblies were dereplicated using dRep [29] with a 95% average nucleotide identity (ANI) threshold (-comp 70 -con 10 -str 100 -strW 0). Non-redundant MAGs were retained with completeness > 70% and contamination < 10% based on CheckM [30].

### Species classification and abundance prediction of MAGs

The taxonomic classification of MAGs was predicted using GTDB-Tk [31] based on the genome taxonomy database (GTDB) [32], and determined by the lowest taxonomic rank. Downstream analyses were conducted through the MAGpy [33], which integrates multiple tools including CheckM [30], Prodigal [34], PfamScan [35], Sourmash [36], PhyloPhlAn [37], and DIAMOND BLASTP [38]. The phylogenetic tree of MAGs was constructed by GTDB-Tk (classify_wf) based on 120 conserved single-copy genes from microbial genomes and drawn by IQ-TREE [39]. The “quant_bins” module based on MetaWRAP [40] was used to evaluate the abundance of MAGs in the metagenome using Salmon [41].

### The function annotation of MAGs

Gene prediction and translation were performed using Prodigal [34], followed by the construction of non-redundant protein catalogs through CD-HIT [42]. Functional characterization involved BLASTp alignments via DIAMOND [38] and eggNOG annotations using eggNOGmapper [43]. Carbohydrate-active enzymes (CAZymes) genes were identified by hmmscan [44] based on the dbCAN database [45]. Virulence genes were annotated using DIAMOND alignment to the VFDB database (70% similarity, e-value < 1e-5) [46]. Antibiotic resistance genes (ARGs) were characterized by RGI based on the CARD database [47]. Secondary metabolite biosynthetic gene clusters (SMBGs) were identified by antiSMASH [48] and displayed by Cytoscape [49]. Pathogen-host interactions (PHI) genes were predicted using TBtools [50] based on the PHI database [51]. The ANI and syntenic analysis between genomes were calculated by FastANI [52] and JCVI [53]. Secretome analysis were predicted by TMHMM [54] and Deeploc [55]. Kyoto encyclopedia of genes and genomes orthology (KO) were annotated by KofamKOALA [56]. The non-metric multidimensional scaling (NMDS) plots were drawn using the metaMDS function. The abundance of gene families and annotation of metabolic pathways were normalized to count per million (CPM). The statistical significance of differences between groups was analysed via two-tailed, unpaired Student’s t-tests. Differential microbial feature analysis across cohorts was conducted using linear discriminant analysis effect size (LEfSe) [57]. The non-parametric factorial Kruskal–Wallis test was used as the initial screening step (*p* < 0.05), followed by linear discriminant analysis (LDA) with a default significance threshold of LDA score > 2.0.

## Results

### Reconstruction of MAGs from fecal metagenomes

**Fig. 1:**
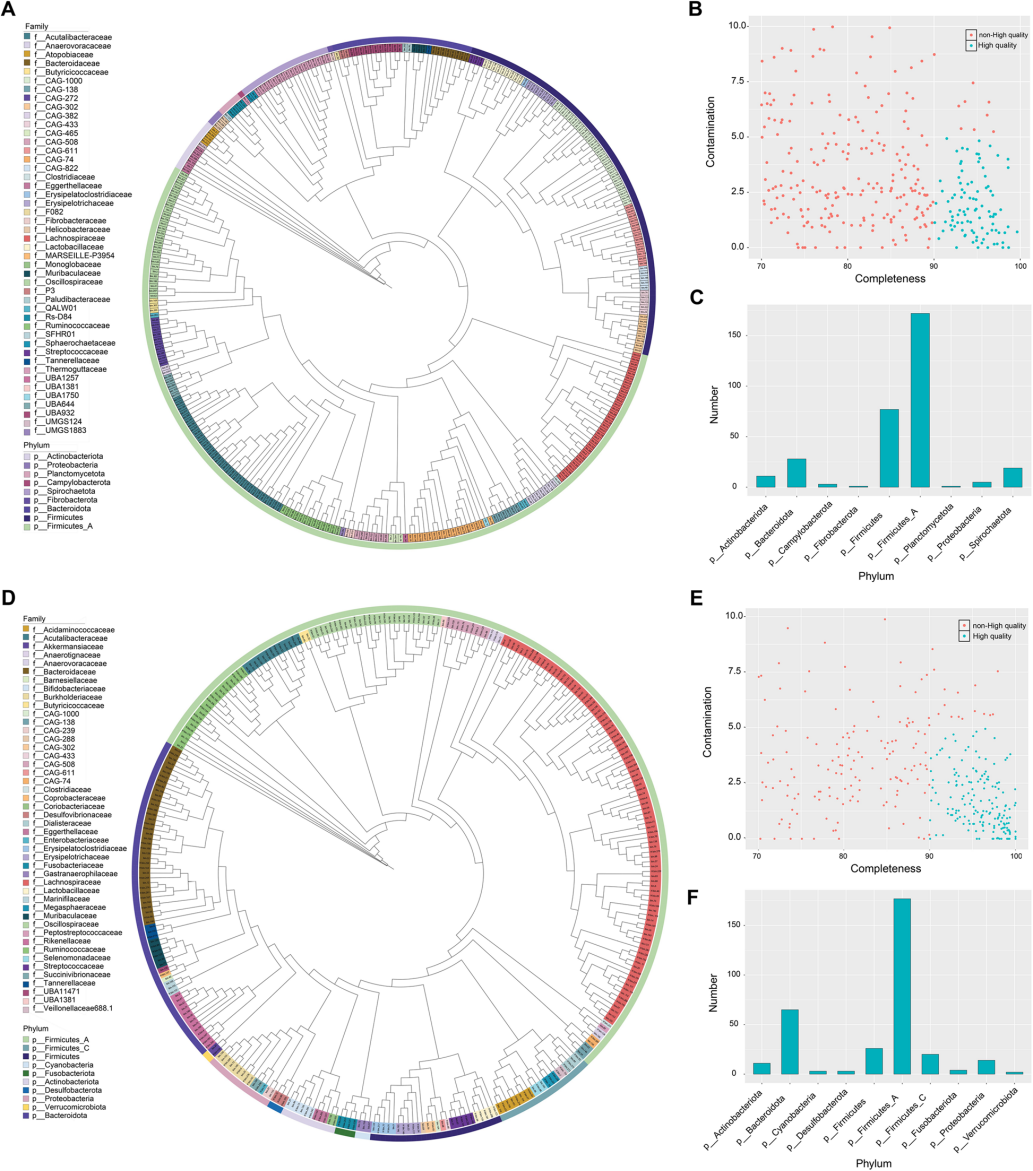
The taxonomic labels of MAGs. **A** The phylogenetic tree of 317 MAGs from fecal metagenomes of macaques. **B** The completeness and contamination of macaque MAGs. The green point showed the MAGs with estimated completeness > 90% and contamination < 5%. **C** The number of 317 macaque MAGs at phylum level. **D** The phylogenetic tree of 325 MAGs from fecal metagenomes of humans. **E** The completeness and contamination of human MAGs. The green point showed the MAGs with estimated completeness > 90% and contamination < 5%. **F** The number of 325 human MAGs at phylum level

A total of 763 non-redundant MAGs were assembled from metagenomic binning of 10 fecal metagenomes of T2DM and asymptomatic macaques. We further obtained 317 MAGs with estimated completeness > 70% and contamination < 10% through co-assembly binning (Fig. 1A). Of these, 96 MAGs with estimated completeness > 90% and contamination < 5%, 41 MAGs had completeness > 95% and contamination < 5%, and 4 MAGs had completeness > 95% without contamination (Fig. 1B). Overall, the 317 MAGs assembled contained 40 to 1,018 contigs, with genome sizes ranging from 0.72 to 3.51 Mb. Each MAG exhibited average contig lengths of 2,037.1 to 45,068.5 bp and encoded 834 to 3,639 protein-coding genes (Table S2). Next, we performed the classification of MAGs in different taxonomic levels through GTDB-Tk [31], and all MAGs were identified at the family level at least. At ANI thresholds of 95%, most of the 317 MAGs could be assigned to known taxonomy (Table 1). At the phylum level, 172 MAGs were defined as Firmicutes_A, followed by Firmicutes (*N* = 77), Bacteroidota (*N* = 28), Spirochaetota (*N* = 19), Actinobacteriota (*N* = 11), Proteobacteria (*N* = 5), Campylobacterota (*N* = 3), which were consistent with the phylogenetic tree (Fig. 1C). Notably, a total of 150 MAGs (47.3%) were regarded as potential novel species, which were not present in the GTDB database. These potential novel species belonged to 11 classes of 9 phyla, mainly including 59 MAGs of class Clostridia, 48 MAGs of class Bacilli, 14 MAGs of class Clostridia_A, 13 MAGs of class Spirochaetia, 6 MAGs of class Bacteroidia, and 5 MAGs of class Coriobacteriia (Table S3).

**Table 1:** The distribution of all macaque metagenome-assembled genomes (MAGs) in different taxonomic levels

Taxonomic level	Classified MAGs	Identified taxa	Unclassified MAGs
Kingdom	317	1	0
Phylum	317	9	0
Class	317	11	0
Order	317	22	0
Family	317	48	0
Genus	285	156	32
Species	167	167	150

We further obtained 325 MAGs with estimated completeness > 70% and contamination < 10% from 20 fecal metagenomes of T2DM and asymptomatic humans (Fig. 1D), of which 79 MAGs were exclusively derived from the guts of T2DM patients. Among them, 167 MAGs with estimated completeness > 90% and contamination < 5%, 110 MAGs had completeness > 95% and contamination < 5%, and 12 MAGs had completeness > 95% without contamination (Fig. 1E). The number of contigs with 325 MAGs obtained by assembly is between 19 and 1,964. The size of each MAG ranged from 0.94 to 6.89 Mb, and the average size of contigs in each MAG ranged from 1914.29 to 84,168.42 bp. Each MAG encoded 1,007 to 6,214 protein-coding genes (Table S4). Further, all MAGs were identified to family level at least (Table 2). At the phylum level, 177 MAGs were defined as Firmicutes_A, followed by Bacteroidota (*N* = 65), Firmicutes (*N* = 26), Firmicutes_C (*N* = 20), Proteobacteria (*N* = 14), Actinobacteriota (*N* = 11), Fusobacteriota (*N* = 4), Cyanobacteria (*N* = 3), Desulfobacterota (*N* = 3), and Verrucomicrobiota (*N* = 2) (Fig. 1F; Table S5).

**Table 2:** The distribution of all human MAGs in different taxonomic levels

Taxonomic level	Classified MAGs	Identified taxa	Unclassified MAGs
Kingdom	325	1	0
Phylum	325	10	0
Class	325	13	0
Order	325	24	0
Family	325	46	0
Genus	324	161	1
Species	307	307	18

### The functional characterization of 317 macaque MAGs and 325 human MAGs

**Fig. 2:**
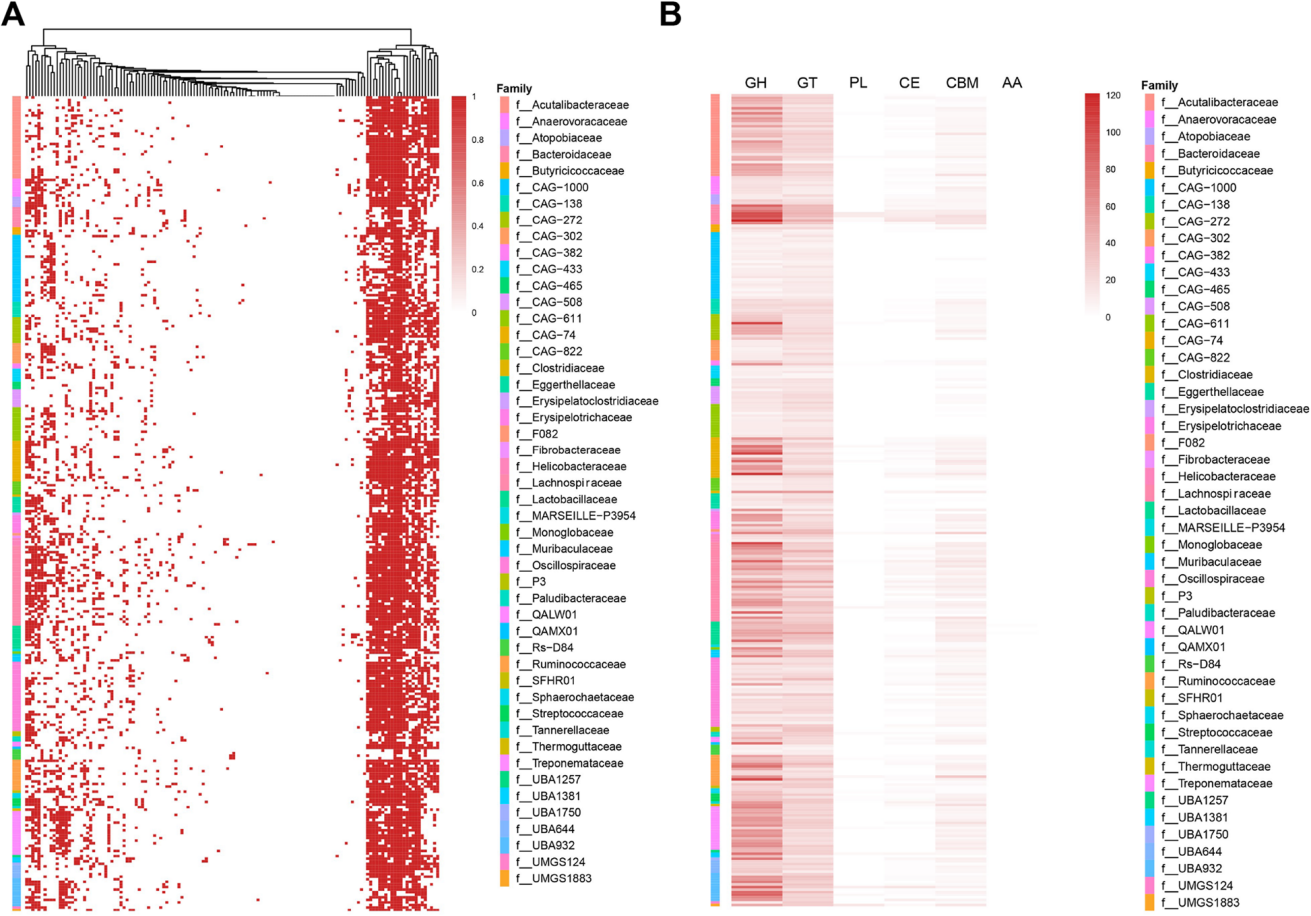
The functional characterization of MAGs. **A** The distribution of virulence genes in macaques MAGs. **B** The distribution of carbohydrate-active enzymes (CAZymes) genes in macaque MAGs. GH: glycoside hydrolases; GT: glycosyl transferases; PL: polysaccharide lyases; CE: carbohydrate esterases; CBM: carbohydrate-binding module; AA: auxiliary activity enzymes

To further compare the functional features, we predicted virulence genes and CAZymes genes in the genomes of each MAG. A total of 116 virulence genes were identified in macaque MAGs and 166 virulence genes in human MAGs (Fig. 2A; Fig. S1A). Notably, MAGs of Lachnospiraceae carried the most abundant virulence-associated genes and the most abundant CAZymes genes in both macaque and human MAGs. Key enzyme families glycoside hydrolases (GH), carbohydrate esterases (CE), carbohydrate-binding modules (CBM), and glycosyltransferases (GT) predominantly clustered within the family Lachnospiraceae (Fig. 2B; Fig. S1B). Comparative analysis of GH family distribution revealed that GH1, GH2, GH3, GH13, GH31, and GH77, involved in glycogen degradation and oligosaccharide processing, were significantly enriched in Lachnospiraceae MAGs from both macaques and humans (Fig. S2).

To investigate the function of potentially novel species, we compared KOs between potentially novel and known species. It illustrated that genes encoding type IV pilus assembly proteins, acetate kinase, and DNA repair enzymes were enriched in the potentially novel species, indicating that these species may provide new insights into motility, short-chain fatty acid production, and environmental adaptation (Fig. S3A). We carried out extensive mining of SMBGs and explored 715 SMBGs from 317 macaque MAGs, 906 SMBGs from 325 human MAGs (Table S6). By matching with the MiBIG database, we identified 175 matched BGCs in macaque MAGs and 223 in human MAGs. The largest number of biosynthetic gene clusters (BGCs) were astallatene (BGC0002397.2), freyrasin (BGC0002306.2), and sodorifen (BGC0002283.2). We found that Lachnospiraceae has great potential to produce astallatene, freyrasin, and sodorife. 9.30% of the regions, especially in the potential novel species, exhibited no match in the MiBIG database, indicating that the structure and function have not yet been described (Fig. S3B; Table S6).

### Characterization of gut microbiome between T2DM and healthy groups

**Fig. 3:**
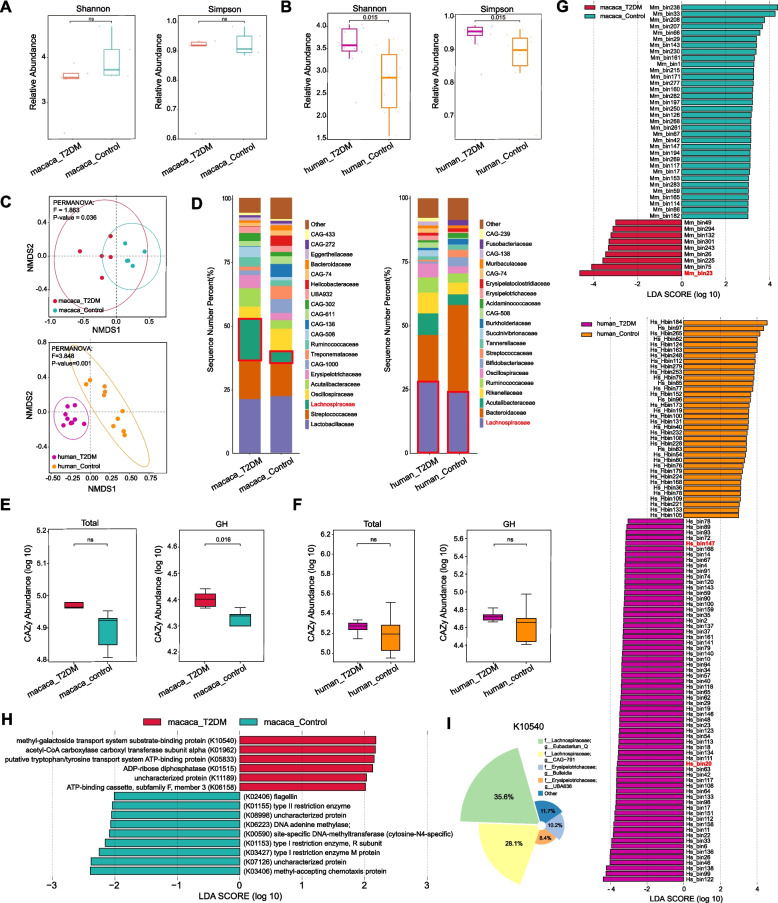
The abundance comparison of MAGs in fecal metagenomes of T2DM and control groups. **A** The alpha diversity (Shannon index and Simpson index) of 317 macaque MAGs (ns, not significant, two-tailed t-test). **B** The alpha diversity (Shannon index (*p* = 0.015, two-tailed t-test) and Simpson index (*p* = 0.015, two-tailed t-test)) of 325 human MAGs. **C** Non-metric multidimensional scaling (NMDS) analysis of T2DM and control groups. **D** The main gut microbiota composition at the family level. **E** Differential analysis of CAZymes in macaque T2DM and control groups (Total: ns, not significant, two-tailed t-test; GH: *p* = 0.016, two-tailed t-test). **F** Differential analysis of CAZymes in human T2DM and control groups (Total: ns, not significant, two-tailed t-test; GH: ns, not significant, two-tailed t-test). **G** Linear discriminant analysis effect size (LEfSe) analysis between T2DM and control groups. **H** The significant differences of kyoto encyclopedia of genes and genomes orthology (KO) between macaque T2DM and control groups. **I** The contribution of macaque MAGs in K10540 term

Based on the abundances of 317 macaque MAGs and 325 human MAGs, we compared differences in gut microbiome between T2DM and control groups at the genomic level. The α-diversity of MAGs, including Shannon and Simpson indices, showed no significance between macaque T2DM and control groups, but was significantly higher in the human T2DM group than in the control group (*p* < 0.01) (Fig. 3A, B). NMDS analysis revealed that T2DM macaques and T2DM humans had significant separation of microbial communities from control groups (*p* < 0.05) (Fig. 3C). In particular, the proportion of Lachnospiraceae was obviously more abundant in the guts of both T2DM groups (Fig. 3D). CAZyme showed increased abundance in both macaque and human T2DM microbiomes. Specifically, GH family abundance was significantly elevated in T2DM macaques (*p* < 0.05), showing consistent enrichment trends in humans, predominantly driven by Lachnospiraceae (Fig. 3E, F; Fig. S2; Fig. S4A).

We compared the relative abundance of gut bacteria between T2DM and control groups using LEfSe [57] (Fig. 3G). The abundances of 43 macaque MAGs exhibited significant differences, with 9 MAGs (Mm_bin23 (Lachnospiraceae), Mm_bin75 (Lachnospiraceae), Mm_bin225 (Ruminococcaceae), Mm_bin26 (Acutalibacteraceae), Mm_bin243 (UBA932), Mm_bin301 (Erysipelotrichaceae), Mm_bin132 (CAG-508), Mm_bin294 (Atopobiaceae), and Mm_bin49 (Ruminococcaceae)) significantly more abundant in the guts of T2DM macaques (*p* < 0.05 and LDA > 2.5). Among them, Mm_bin23, belonging to genus *Eubacterium_Q*, exhibited the most pronounced alterations (*p* < 0.01 and LDA > 4). LEfSe analysis identified 66 human MAGs with significantly increased abundance in the T2DM patients (*p* < 0.05 and LDA > 2.5). The majority of these enriched MAGs were affiliated with the family Lachnospiraceae (*N* = 20), of which two members of *Eubacterium*, the Hs_bin20 (*Eubacterium_F*) and Hs_bin147 (*Eubacterium_I*), were scarcely detected in the fecal metagenomes of control groups but were prevalent in fecal metagenomes of T2DM groups (Table S4). Specifically, MAGs in both T2DM groups exhibited enrichment of genes associated with carbohydrate uptake (macaque: K10540), sugar transport (human: K02057), lipid biosynthesis (macaque: K01962), and bile acid metabolism (human: K07007) (Fig. 3H; Fig. S4B). Taxonomic attribution of significantly enriched KOs revealed that T2DM-enriched KOs were primarily associated with members of the Acutalibacteraceae in humans, and in macaques, mainly associated with members of Lachnospiraceae, particularly the genus *Eubacterium_Q*, which was the major contributor to K10540. (Fig. 3I; Table S7).

### Potential microbes associated with T2DM

**Fig. 4:**
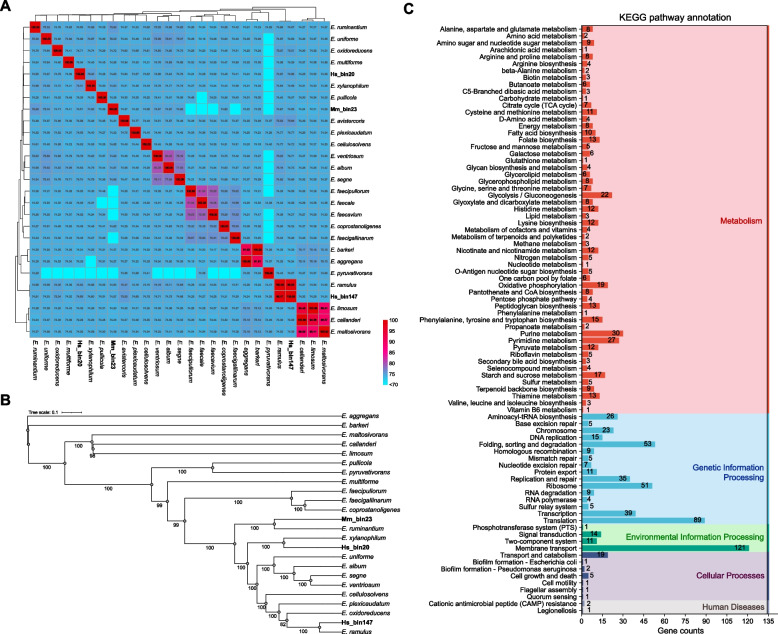
The genomes of Mm_bin23, Hs_bin20, and Hs_bin147. **A** Average nucleotide identity (ANI) heatmap. **B** The phylogenetic tree of *Eubacterium.* **C** KEGG pathway annotation of Mm_bin23

To further investigate microbes associated with T2DM, we focused on the genus *Eubacterium* due to its significant abundance changes and prominent roles in KO functional profiles. To investigate the genetic relationship of the three T2DM-enriched *Eubacterium* MAGs, we performed comparative genomic analyses using 24 *Eubacterium* genomes downloaded from the NCBI (Table S8). Hs_bin147 exhibited the highest ANI (> 99.17%) with *E. ramulus*, while Mm_bin23 and Mm_bin147 showed no significant matches (ANI < 80%) to any other species (Fig. 4A). Phylogenetic reconstruction using IQ-TREE [39] with 120 single-copy marker genes from the GTDB-TK database demonstrated that all three MAGs clustered within GTDB-defined genus-level clades. Mm_bin23 (*Eubacterium_Q sp900314445*) grouped with *E. ruminantium* in the *Eubacterium_Q* clade, Hs_bin20 (*Eubacterium_F sp003491505*) aligned with *E. xylanophilum* in the *Eubacterium_F* clade, and Hs_bin147 formed a lineage with *E. ramulus* in the *Eubacterium_I* clade (Fig. 4B). The syntenic analysis further supported the taxonomic assignment of Hs_bin147, showing high synteny of contigs with the *E. ramulus* genome, consistent with the MAGs annotation results (Fig. S5). Functional annotation of Mm_bin23 predicted 1,773 protein-coding genes, with 1,545 (87.14%), 38 (2.14%), and 1,020 (57.53%) genes annotated by COG, GO, and KEGG databases, respectively (Fig. 4C; Fig. S6). In addition, secretome analysis integrating TMHMM [54] and Deeploc [55] predictions identified 42 putative secreted proteins, including 9 Type III secretion system effector proteins (Table S9). Meanwhile, a total of 2,662 and 2,548 protein-coding genes were identified from Hs_bin20 and Hs_bin147. Hs_bin20 harbored 2,298 COG-annotated genes (86.3%), 37 GO-annotated genes (1.4%), and 1,406 KEGG-annotated genes (52.8%) (Fig. S7). Hs_bin147 was characterized by 2,266 COG-annotated genes (89.0%), 23 GO-annotated genes (0.9%), and 1,471 KEGG-annotated genes (57.7%) (Fig. S8).

**Fig. 5:**
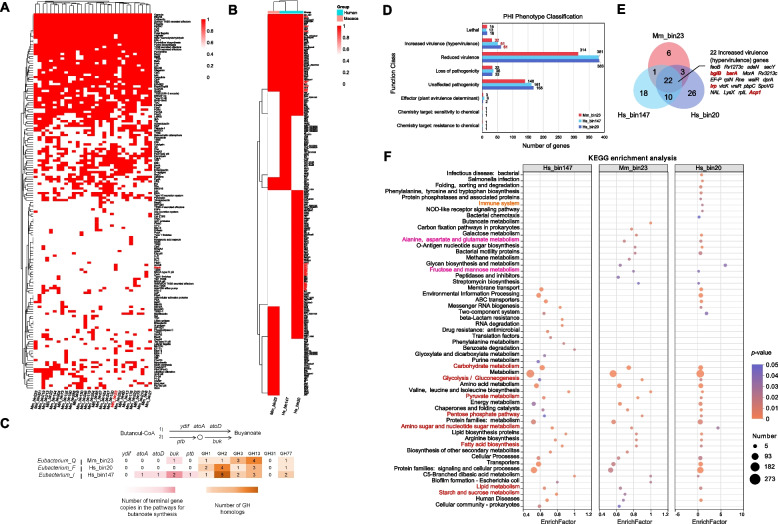
Functional analysis of Mm_bin23, Hs_bin20, and Hs_bin147. **A** The annotation of virulence genes in genomes of Lachnospiraceae from macaques MAGs. **B** The annotation of virulence genes in genomes of Mm_bin23, Hs_bin20, and Hs_bin147. **C** Heatmap of core genes in the butanoate synthesis pathway and GH familes. **D** Pathogen host interactions (PHI) classification in Mm_bin23, Hs_bin20, and Hs_bin147. **E** Venn plot of increased virulence (hypervirulence) genes. **F** KEGG enrichment of PHI genes

To functional analysis of the genomes of the three microbes associated with T2DM, we reconstructed 31 MAGs of Lachnospiraceae from macaque samples, with 24 MAGs assigned to the species level (Table S3). Virulence gene annotation based on the Lachnospiraceae MAGs revealed that genes associated with BapA were exclusively identified in Mm_bin23 (Fig. 5A). Furthermore, comparative analysis with other two MAGs of *Eubacterium* (Hs_bin20 and Hs_bin147) showed that the virulence genes shared by all three MAGs were associated with secretion systems, including Type III secretion system (TTSS) (*cdsN*), Type VII secretion system (*essC*), and TTSS secreted effectors (*glgA*, *yycJ*) (Fig. 5B). Genes predicted to encode secreted toxin modules (HlyA: *hlyB*, Cereulide: *cesC*, Colibactin: *clbM*) and mucosal attachment module (Type IV pili: *pilT*) were also detected in these MAGs. Moreover, the virulence genes of Hs_bin20 were enriched in flagella-associated genes, such as *cheV*, *flaA*, *flgC*, *flgD*, *flgE*, *flgF*, *flgG*, *flgK*, *fliA*, *fliM*, *fliN*, *fliP*, and *fliR*. Given that several members of Lachnospiraceae exhibited intestinal butyrate production ability, we further investigated genes related to butyrogenesis pathways in the three MAGs. Two distinct enzymatic routes convert butanoyl-CoA to butanoate, including the butyrate kinase pathway (*buk* and *ptb*) and the acetate CoA transferase pathway (*ydif*, *atoA*, and *atoD*). (Table S10). It revealed that Mm_bin23 and Hs_bin20 lacked essential genes for the dominant acetate CoA-transferase and butyrate kinase pathways of butyrate synthesis, suggesting a potential loss of butyrogenic capability. All three *Eubacterium* MAGs exhibiting significantly increased abundance in the T2DM group harbored gene copies encoding glycoside hydrolases implicated in oligosaccharide and starch degradation, specifically GH1, GH2, GH3, GH13, and GH77 (Fig. 5C).

The genes related to PHI are important factors. To assess the pathogenicity of the three MAGs, a total of 1,854 genes were annotated using the PHI database [51], with 32, 51, and 61 genes classified as increased virulence (hypervirulence) genes in Mm_bin23, Hs_bin147, and Hs_bin20, respectively (Fig. 5D). Notably, all three MAGs harbored hypervirulence genes associated with diabetes, including *bglB*, *barA*, *lrp*, and *Acp1* (Fig. 5E). KEGG enrichment of PHI genes revealed significant enrichment in diabetes-related pathways across the MAGs (Fig. 5F). Specifically, carbohydrate metabolism, glycolysis/gluconeogenesis, fatty acid biosynthesis, lipid metabolism, amino acid metabolism, starch and sucrose metabolism, and pyruvate metabolism pathways were commonly enriched in the three MAGs. In addition, Mm_bin23 and Hs_bin20 uniquely shared enrichment in alanine, aspartate, and glutamate metabolism and fructose and mannose metabolism, while Hs_bin20 exclusively exhibited enrichment in the immune system pathway.

## Discussion

Microbial genomes enable deeper insights into gut microbiome functions in host metabolism and disease. Reconstructing genomes from metagenomic data could expand the catalog and diversity of available microbial genomes, particularly for uncultivable strains [58, 59]. While studies have characterized the gut microbial composition in T2DM macaques, genomic-level understanding remains limited, especially in spontaneous diabetic individuals [16, 20, 60, 61]. We reconstructed a total of 317 MAGs and 325 MAGs from macaque and human fecal metagenomes, respectively. About 75% of these MAGs belonged to phyla Firmicutes_A, Firmicutes, and Bacteroidota, which accorded with previous studies based on metagenomic sequencing or 16S rRNA sequencing [16, 20, 62–64]. Compared with reference genomes of GTDB, almost half of the macaque MAGs were potential novel species, primarily belonging to classes Clostridia and Bacilli, which played key roles in the maintenance of overall gut function [65, 66]. Functional annotation revealed that these potential novel species were significantly enriched in pathways related to environmental stress response, cell wall synthesis and remodeling, transport systems, motility, and metabolism, suggesting adaptations that may enhance their fitness and competitive advantage within the gut niche.

Co-assembly proved particularly effective for recovering both high- and low-abundance microbial MAGs, which increased the yield of novel taxa. MAGs based differential analysis revealed significantly increased gut microbial diversity in T2DM patients, whereas no significant difference between T2DM macaques and controls. This may be attributed to the standardized captive environments of macaques minimizing external influences compared to humans with diverse lifestyles [67]. Moreover, the inherent heterogeneity in human lifestyles and clinical histories, including diet, BMI, ethnicity, and disease duration, necessitates large-scale, deeply phenotyped longitudinal studies to fully disentangle these confounding factors. The Lachnospiraceae species are recognized as key contributors to metabolic modulation, their functional roles in the gut remain debated and might influence healthy functions [68]. Recent studies demonstrated that insulin-resistant patients exhibit increased fecal monosaccharides associated with Lachnospiraceae [69]. Furthermore, Lachnospiraceae was significantly enriched in the gut microbiota of T2DM macaques, which may promote fatty acid β-oxidation disorders [16]. Similarly, Lachnospiraceae MAGs showed significant abundance differences between T2DM macaques/patients and asymptomatic individuals in our study. The enriched KOs and CAZymes suggested that the changes in sugar transport function and fatty acid synthesis in the T2DM group were mainly associated with the family of Lachnospiraceae. Their significant enrichment in T2DM hosts, coupled with altered functional profiles, suggested conserved roles across primate intestinal microbiomes, potentially influencing host physiology, regulating gut ecological balance by secondary metabolites, and driving dysregulation of fatty acid metabolism [16, 69, 70].

In macaque-derived MAGs of Lachnospiraceae, Mm_bin23 belonged to *Eubacterium*, exhibited the most pronounced abundance increase in T2DM macaques, and contained high diversity of virulence determinants, including unique adherence-associated virulence factor BapA. As a biofilm-associated protein, BapA promotes persistent colonization by enhancing adhesion and biofilm formation, thereby potentially exacerbating gut barrier dysfunction and inflammation [71, 72]. Meanwhile, we identified two significantly enriched *Eubacterium* MAGs, Hs_bin20 and Hs_bin147, from human T2DM patients. The co-occurrence of specific virulence genes in Mm_bin23, Hs_bin20, and Hs_bin147 carried determinants for capsule biosynthesis (Capsule), encoding major surface structures of immune evasion and barrier integrity [73, 74]. In addition, shared virulence factors included adhesion systems such as Type IV pili and toxin production such as HlyA. These adherence-associated factors facilitate mucosal attachment and biofilm formation [75], which may promote chronic irritation of the gut mucosa. Simultaneously, the production of toxins can disrupt epithelial barrier integrity, potentially exacerbating inflammation and the translocation of pro-inflammatory microbial products [76]. This process may further trigger insulin resistance through the activation of inflammatory signaling pathways. Specifically, genes associated with flagella and chemotaxis were abundantly identified in Hs_bin20, suggesting that this MAG possesses enhanced potential for motility and adhesion [77]. These multifaceted virulence-associated genes underscored the potential association of *Eubacterium* with T2DM in exacerbating intestinal barrier dysfunction and inflammation. Further analysis of the pathogen-host interactions of MAGs revealed that PHI genes carried by Mm_bin23, Hs_bin20, and Hs_bin147 genomes were significantly associated with T2DM-related pathways, especially core carbohydrates, lipid, and amino acid metabolism. Moreover, diabetes-associated genes, including *bglB*, *barA*, *lrp*, and *Acp1*, were identified within the hypervirulence genes of all three MAGs. *bglB* is a β-glucosidase encoding gene that plays a crucial role in the breakdown of polysaccharides into glucose. The hydrolytic activity of β-glucosidase was gradually inhibited with increasing glucose concentrations [78]. *barA* encodes sensor kinases in two-component systems that enhance virulence gene expression, potentially increasing gut permeability by activating factors such as adhesins, thereby contributing to chronic inflammation [79]. The *lrp* gene influences leucine metabolism and modulates the gut microbial production of branched-chain amino acids (BCAAs) [80]. Elevated BCAA levels are associated with insulin resistance [81]. *Acp1* encodes an acid phosphatase implicated in regulating insulin signaling pathways and lipid metabolism [82]. These MAGs of *Eubacterium* were scarcely detected in the fecal metagenomes of control groups but were prevalent in the fecal metagenomes of T2DM groups. The similar virulence-associated genes and diabetes-associated host-interaction factors between macaque-derived and human-derived MAGs of *Eubacterium* underscored the potential role of these bacteria in the pathogenesis of T2DM. Virulence factors could exacerbate inflammation and barrier dysfunction, while metabolic interference with enhanced glucose liberation and promotion of insulin resistance potentially fuels the metabolic dysregulation characteristic of T2DM. This pattern exhibited the shared genomic features of *Eubacterium* in both T2DM hosts, which require future experimental validation to confirm the causal link.

The species of Lachnospiraceae, in particular *Eubacterium*, are increasingly recognized as critical regulators in energy homeostasis, colonic motility, immunomodulation, and inflammation [83, 84]. Several species of *Eubacterium* are currently regarded as promising targets for microbial therapeutics [83]. Such observations may be confounded by the complex taxonomic history of *Eubacterium* [5]. Several species initially assigned to this genus have been reclassified into novel or existing genera, such as *Agathobacter rectalis* and *Anaerobutyricum hallii* [84]. Moreover, contrasting with known beneficial effects of butyrate-producing taxa, bidirectional mendelian randomization analysis in a dutch cohort revealed that increased abundance of *Eubacterium* was associated with elevated type 2 diabetes risk [85]. In our study, Mm_bin23 was classified as *Eubacterium_Q sp900314445*, and Hs_bin20 was classified as *Eubacterium_F sp003491505*, awaiting formal taxonomic description. *Eubacterium_F sp003491505* was found at significantly higher abundance in the guts of high-fat diet-fed rats compared to control-fed rats [86]. Comparative genomic analysis confirmed that Hs_bin147 was classified as *E. ramulus*. *E. ramulus* ferments glucose to butyrate, a major energy source of colonocytes, and it has systemic effects influencing insulin resistance [87, 88]. Reportedly, *E. ramulus* was related to fasting blood glucose and endothelial cell function indicators in diabetic patients [89]. Amplicon sequence variants corresponding to *E. ramulus* exhibited significant associations with diabetes status [90]. The elevated abundance of *E. ramulus* in untreated T2DM patients reflected host-microbiome interactions in disease states. Specifically, Mm_bin23 and Hs_bin20 lacked essential genes for acetate-to-butyrate conversion, suggesting a potential shift in metabolic capacity of canonical short-chain fatty acids (SCFAs) production. In addition, enhanced capacity to hydrolyze dietary and glycans potentially diverted SCFA precursors and generated alternative metabolites implicated in gut barrier dysfunction. Future studies employing targeted metabolomics are essential to quantify the actual metabolic fluxes and confirm the proposed mechanisms. Consequently, *Eubacterium* species may be associated with the dysregulation of glucose metabolism in T2DM and represent a novel microbial signature of diabetes that warrants further investigation. The involvement of *Eubacterium* species in the development of T2DM is less clear. While genomic data provided associative evidence and functional potential, further in vivo and in vitro studies are warranted to establish the causal impact of these *Eubacterium* strains on host intestinal homeostasis. Understanding how *Eubacterium* is involved in diabetes development may promote the advancement of novel therapeutic strategies targeting the gut microbiota to improve glycemic control and reduce the risk of insulin resistance.

## Conclusions

In conclusion, through de novo assembly of 317 macaque-derived and 325 human-derived MAGs, we identified conserved potential pathogenic bacteria across species and expanded the genomic repository for primate gut microbiota. Notably, we observed a consistent enrichment of Lachnospiraceae members and *Eubacterium* strains in T2DM hosts, which may contribute to disease progression through carrying intestinal barrier-associated virulence genes and glucose homeostasis-disrupting genes. The spontaneous T2DM macaque model, unbiased by glucose-lowering medications, offers a powerful and translationally relevant platform for elucidating gut microbiome-mediated disease mechanisms and evaluating novel therapeutic strategies. These findings provided an important basis for future research on the pathogenesis and intervention strategies of T2DM.

## Supplementary Information

Additional file 1. Fig. S1 (A) The abundance of virulence genes in macaque and human MAGs. (B) The abundance of CAZymes genes in macaque and human MAGs. Fig. S2 The distribution of glycoside hydrolases (GH) families in macaque and human MAGs. Fig. S3 (A) The significant differences of KOs between potentially novel and known species. (B) Network of the relationship between families and known secondary metabolites in macaque and human MAGs. Fig. S4 (A) Differential analysis of CAZy enzymes in T2DM and control groups. CBM: carbohydrate-binding module; GT: glycosyl transferases; PL: polysaccharide lyases; AA: auxiliary activity enzymes; CE: carbohydrate esterases. (B) The significant differences of KOs between human T2DM and control groups. Fig. S5 Syntenic analysis between Hs_bin147 and *E. ramulus*. Fig. S6 GO pathway annotation and COG function classification of Mm_bin23. Fig. S7 KEGG and GO pathway annotation and COG function classification of Hs_bin20. Fig. S8 KEGG and GO pathway annotation and COG function classification of Hs_bin147.

Additional file 2. Table S1. The summary of information of macaques. Table S2. The characterization of completeness, contamination and genomes of macaque MAGs. Table S3. The taxonomy labels of macaque MAGs annotated by GTDB-TK. Table S4. The characterization of completeness, contamination and genomes of human MAGs. Table S5. The taxonomy labels of human MAGs annotated by GTDB-TK. Table S6. The explored SMBGs. Table S7. Significantly enriched KO terms in T2DM and control groups from macaque and human MAGs (Other: represents the sum of contributions from taxonomic groups that individually accounted for less than 1% of the total contributions.). Table S8. The genomes of *Eubacterium*.Table S9. Genomic characterization of Mm_bin23. Table S10. The core genes in the butanoate metabolism pathways.

## Data Availability

The raw data of macaques analysed during the current study are available in the Genome Sequence Archive (GSA) of the National Genomics Data Center (NGDC), China National Center for Bioinformation, under accession number PRJCA021499 (GSA: CRA013607, https://ngdc.cncb.ac.cn/gsa/browse/CRA013607) (Download: https://download.cncb.ac.cn/gsa4/CRA013607/). The raw data of humans analysed during the current study are available in the Sequence Read Archive (SRA) of the National Center for Biotechnology Information (NCBI) under accession numbers PRJNA361402 (https://www.ncbi.nlm.nih.gov/bioproject/PRJNA361402) and PRJNA638403 (https://www.ncbi.nlm.nih.gov/bioproject/PRJNA638403).

## Abbreviations

T2DM: Type 2 diabetes mellitus
MAG: Metagenome-assembled genome
CAZymes: Carbohydrate-active enzymes
ANI: Average nucleotide identities
GTDB: Genome taxonomy database
ARG: Antibiotic resistance gene
SMBG: Secondary metabolite biosynthetic gene cluster
PHI: Pathogen-host interactions
KO: Kyoto encyclopedia of genes and genomes orthology
NMDS: Non-metric multidimensional scaling
CPM: Count per million
LEfSe: Linear discriminant analysis effect size
LDA: Linear discriminant analysis
CBM: Carbohydrate-binding module
GT: Glycosyl transferases
PL: Polysaccharide lyases
AA: Auxiliary activity enzymes
GH: Glycoside hydrolases
CE: Carbohydrate esterases
SCFA: Short-chain fatty acid
