# Supplementary material for: Metagenome-assembled genomes from the gut microbiome of spontaneous diabetic macaques provide insights into microbes associated with type 2 diabetes mellitus
Source: BMC Microbiol. 2026 Mar 4;26:331. doi: 10.1186/s12866-026-04902-2 (PMC13067670; doi:10.1186/s12866-026-04902-2)
Supplement: Supplementary file 1 — Additional file 1. Fig. S1 (A) The abundance of virulence genes in macaque and human MAGs. (B) The abundance of CAZymes genes in macaque and human MAGs. Fig. S2 The distribution of glycoside hydrolases (GH) families in macaque and human MAGs. Fig. S3 (A) The significant differences of KOs between potentially novel and known species. (B) Network of the relationship between families and known secondary metabolites in macaque and human MAGs. Fig. S4 (A) Differential analysis of CAZy enzymes in T2DM and control groups. CBM: carbohydrate-binding module; GT: glycosyl transferases; PL: polysaccharide lyases; AA: auxiliary activity enzymes; CE: carbohydrate esterases. (B) The significant differences of KOs between human T2DM and control groups. Fig. S5 Syntenic analysis between Hs_bin147 and E. ramulus. Fig. S6 GO pathway annotation and COG function classification of Mm_bin23. Fig. S7 KEGG and GO pathway annotation and COG function classification of Hs_bin20. Fig. S8 KEGG and GO pathway annotation and COG function classification of Hs_bin147. [file 12866_2026_4902_MOESM1_ESM.pdf]

Supplementary Figures

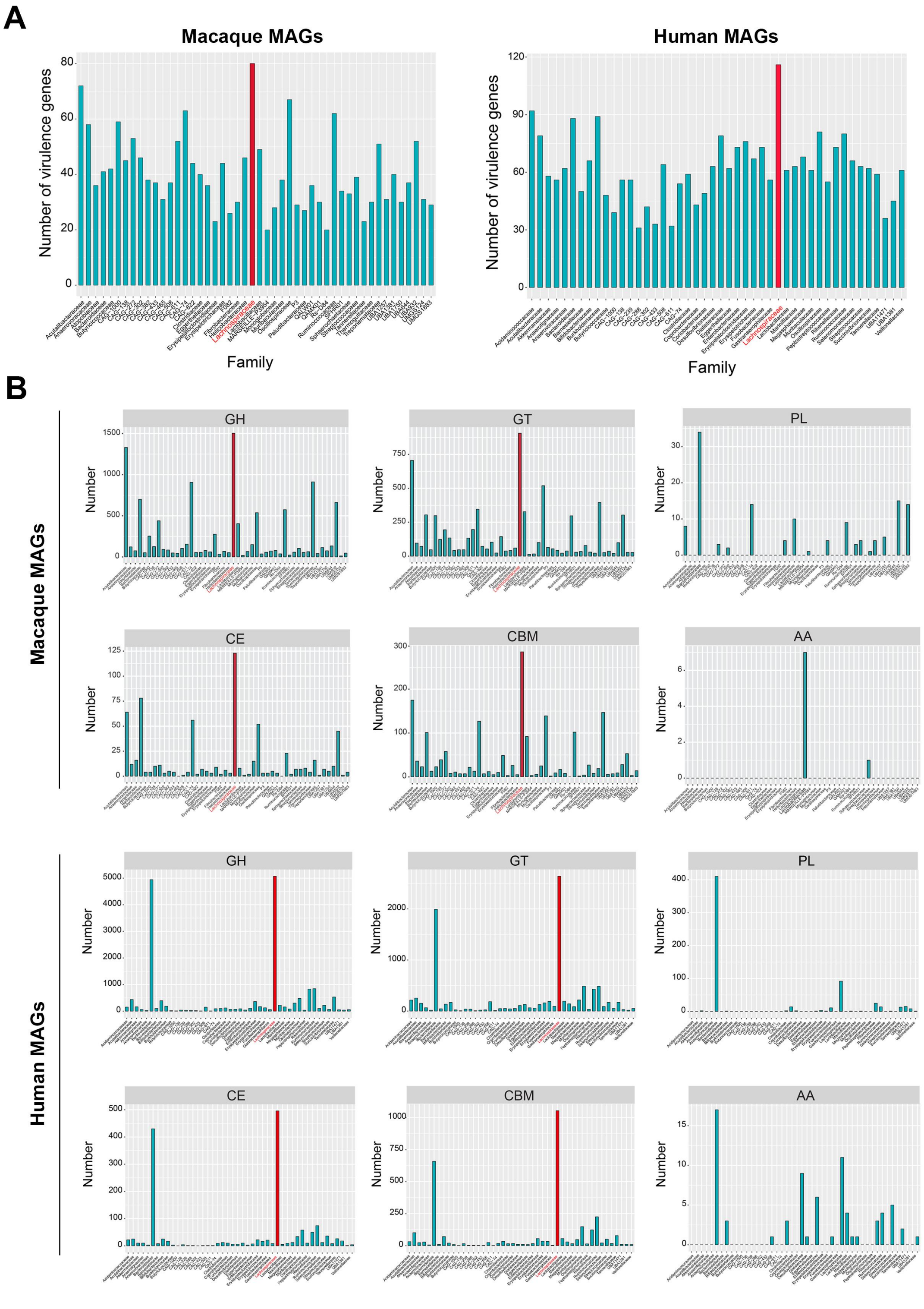

Fig. S1 (A) The abundance of virulence genes in macaque and human MAGs. (B) The abundance of CAZymes genes in macaque and human MAGs.

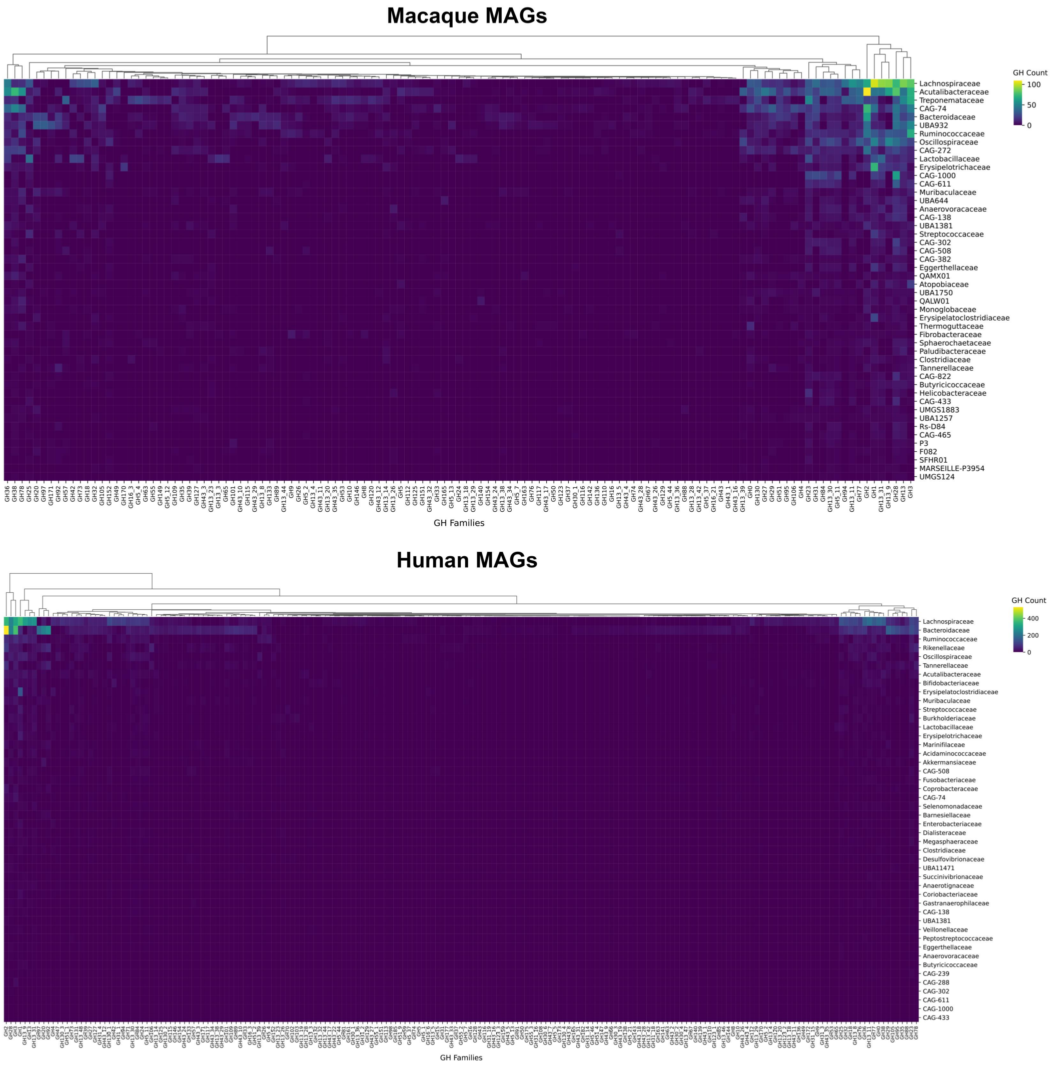

Fig. S2 The distribution of glycoside hydrolases (GH) families in macaque and human MAGs.

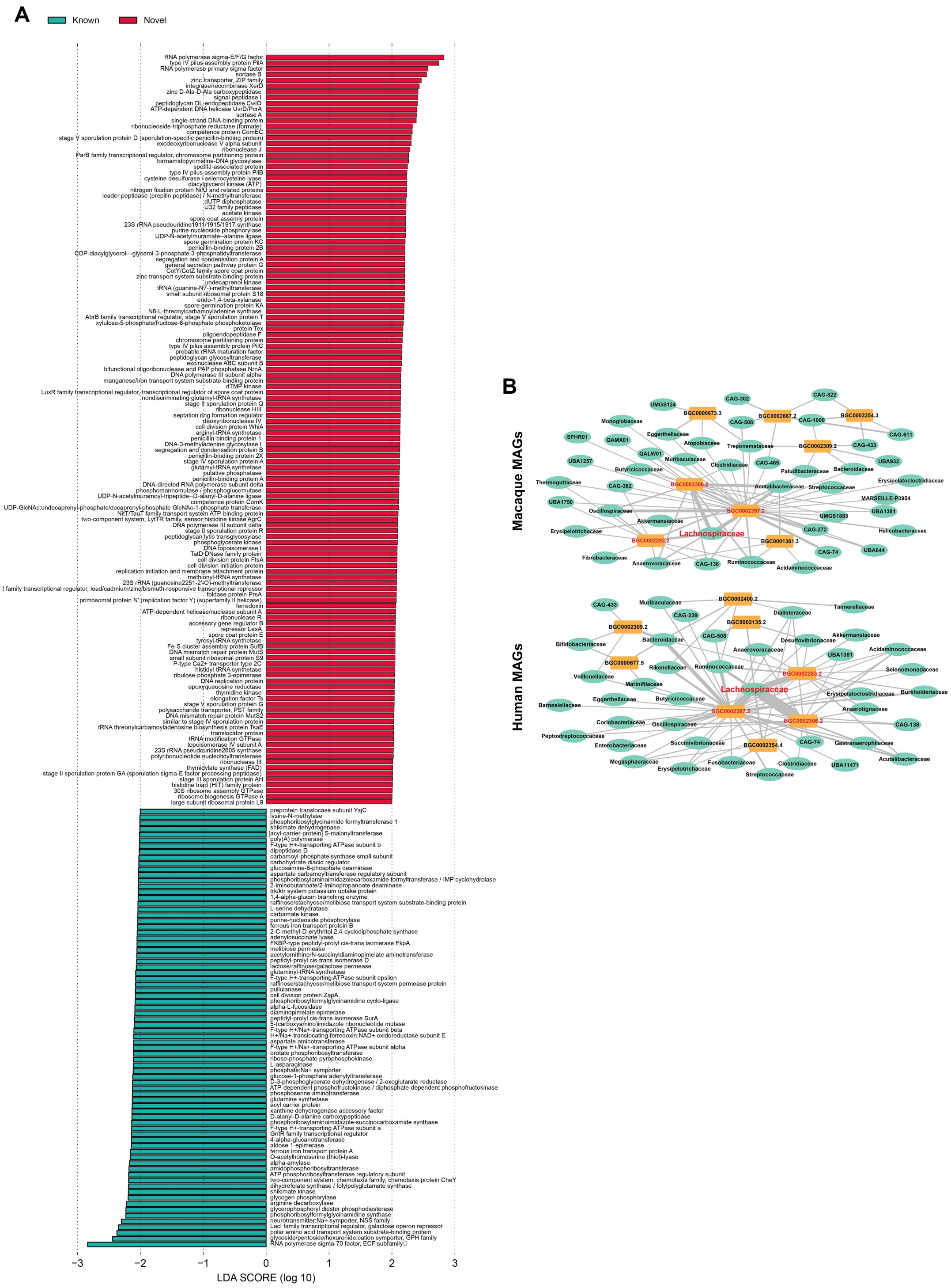

Fig. S3 (A) The significant differences of KOs between potentially novel and known species. (B) Network of the relationship between families and known secondary metabolites in macaque and human MAGs.

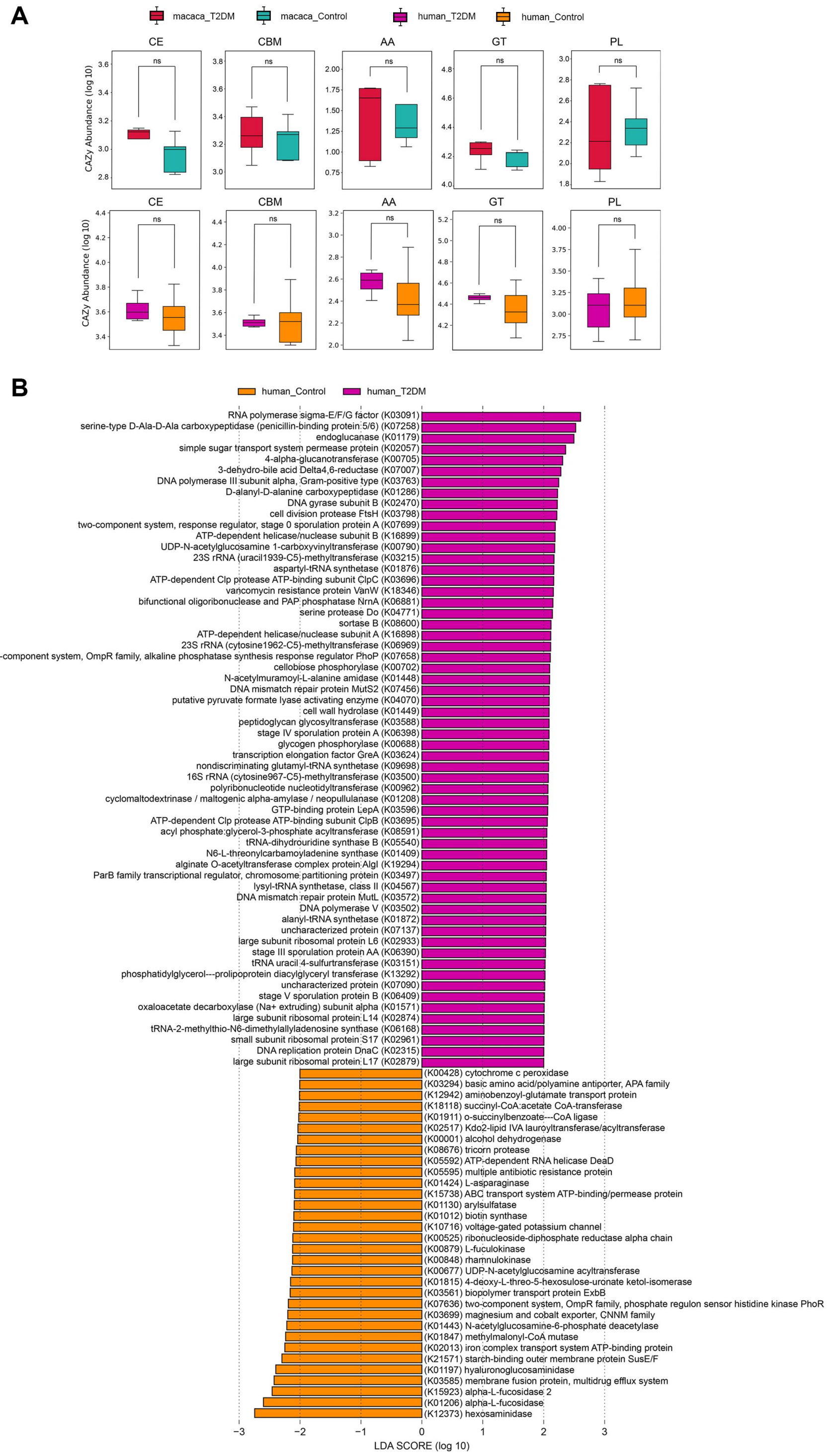

Fig. S4 (A) Differential analysis of CAZy enzymes in T2DM and control groups. CBM: carbohydrate-binding module; GT: glycosyl transferases; PL: polysaccharide lyases; AA: auxiliary activity enzymes; CE: carbohydrate esterases. (B) The significant differences of KOs between human T2DM and control groups.

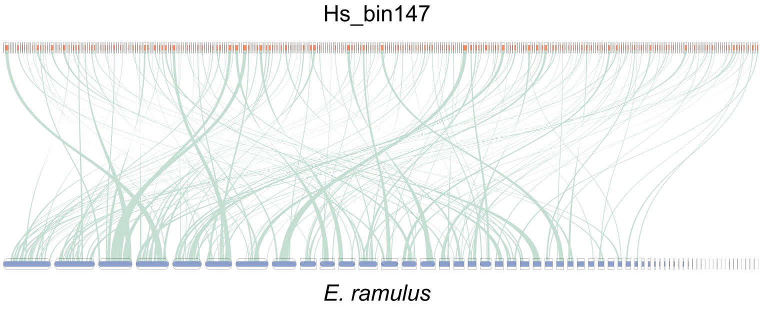

Fig. S5 Syntenic analysis between Hs_bin147 and *E. ramulus*.

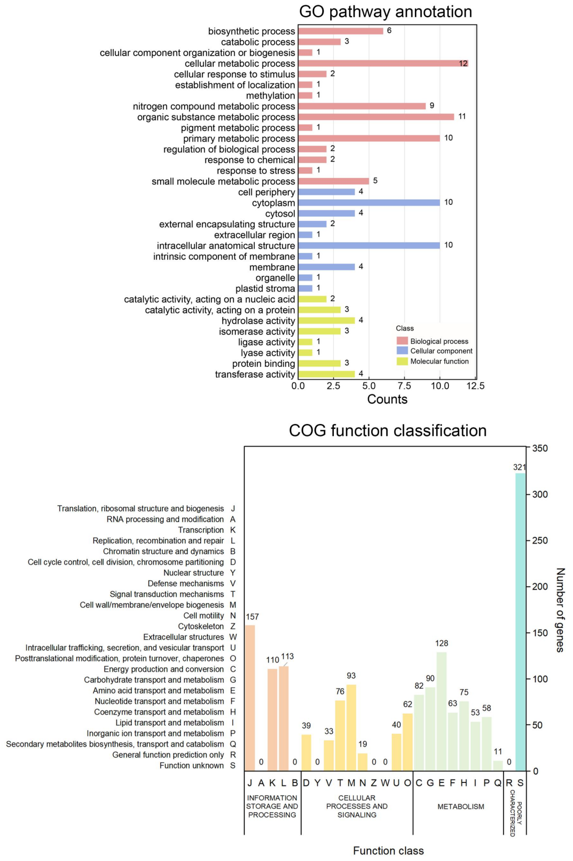

Fig. S6 GO pathway annotation and COG function classification of Mm_bin23.

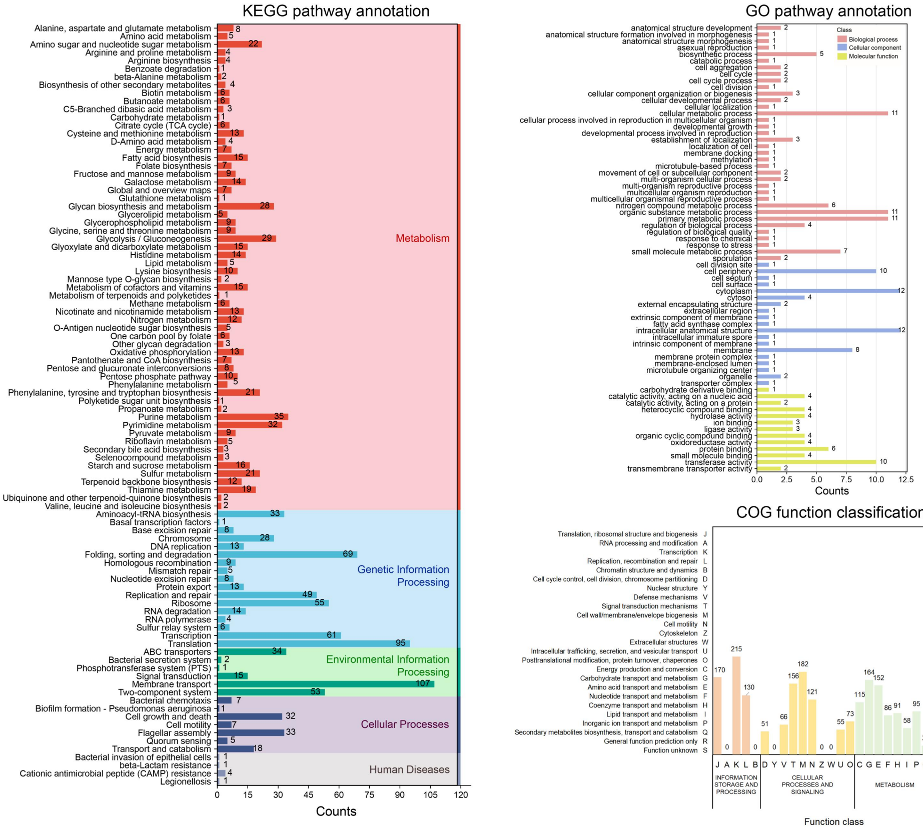

Fig. S7 KEGG and GO pathway annotation and COG function classification of Hs_bin20.

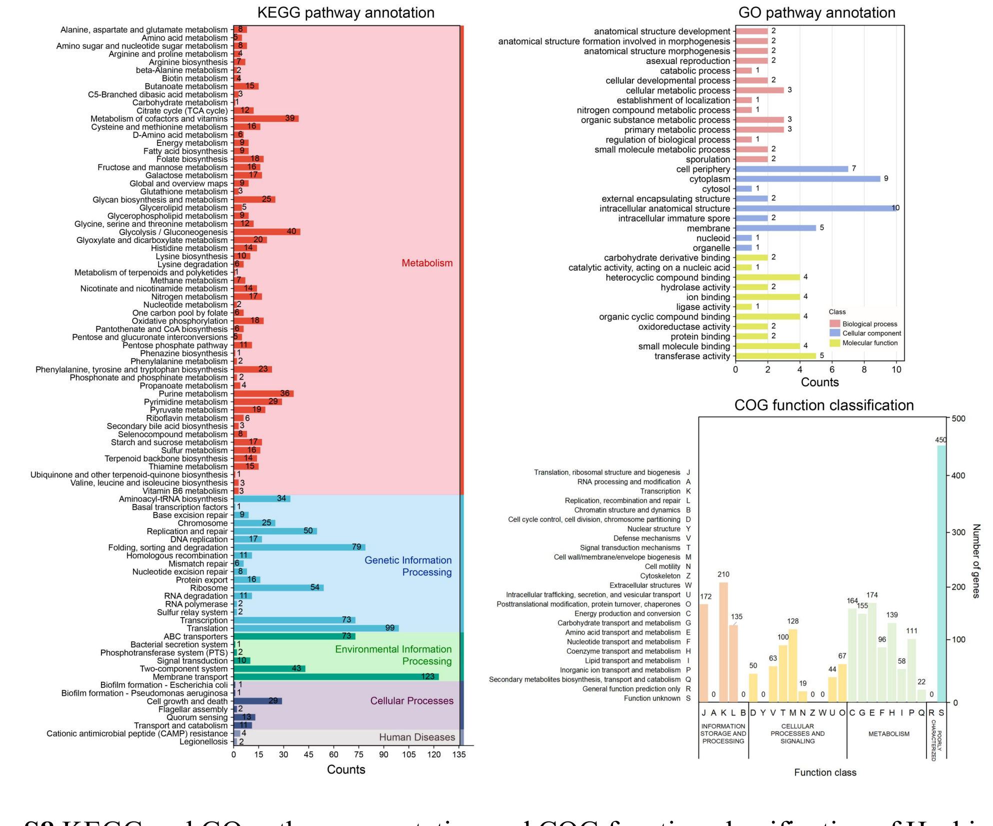

Fig. S8 KEGG and GO pathway annotation and COG function classification of Hs_bin147.
